# Lectures on the Diagnosis and Treatment of Diseases of the Heart

**Published:** 1841-11-13

**Authors:** W. W. Gerhard


### MEDICAL EXAMINER.

DEVOTED TO MEDICINE, SURGERY, AND THE COLLATERAL SCIENCES.

No. 46.] PHILADELPHIA, SATURDAY, NOVEMBER 13, 1841. [Vol. IV.

### LECTURES ON THE DIAGNOSIS AND TREATMENT OF DISEASES OF THE HEART.

BY W. W. GERHARD, M. D.

### LECTURE XVIII.

The *sounds* of the heart furnish us with one of the most important means of diagnosis, but require to be studied in the healthy state before becoming of practical use as signs of disease. The sounds of the heart are two in number, and are designated as first and second sound. The first occurs during the systole or contraction of the heart, and is synchronous with it; it is the longer of the two, and occupies very nearly one-half the whole period of the heart's action, and consequently the first sound may be heard during one-half of the life of each individual. It is described as prolonged but dull, and it may be very readily learned by placing the ear over the heart, while the hand is applied to the pulse of the patient; the vibration of the artery and the sound are heard at the same time, although the sound of the contraction precedes a little the pulsation of the wrist, but the difference is so slight as to be scarcely perceptible. The cause of this sound is differently explained; probably it is not a single cause, but a combination of two, which may in part account for any difference of opinion. The principal cause is certainly the muscular contraction of the heart, which is abundantly capable of producing a sound, as may be verified by experiments upon the hearts of animals. Take the heart of a calf or sheep from the body after sensation has been destroyed, but before the animal is quite dead, and by applying a stethoscope upon it, a sound will be distinctly heard, which is identical with, although weaker than the first sound of the heart; in this there is of course no cause for the sound but pure muscular contraction. But in the living body it is very probable that the sound is in part produced by the friction of the blood against the semilunar valves of both aorta and pulmonary artery; this cause, however, which is not so easy to demonstrate, is by no means so powerful as the muscular contraction. The second sound is the proper valvular sound, and is shown by direct experiment to be caused by the quick contraction of the semilunar valves, especially those of the aorta, which are much stronger than those of the pulmonary artery. If these valves be tied by passing a needle through them in the heart of an animal deprived of sensation, but still living, the second sound is immediately destroyed. The character of the sound is totally different from that of the first; it is very short and sharp, and is properly designated by the term clacking. It follows immediately after the first, and is synchronous with the diastole of the heart; when the semilunar valves are diseased, or prevented from acting by the excessive turgescence of the heart with blood, the second sound is weakened or destroyed. After the second sound, a period of repose, occupying nearly one-half of the time of each complete action of the heart, succeeds, and is again followed by the first sound.

The sounds of the heart are, as may readily be seen, very regular in their succession and proportion, and when these are deranged, a disturbance of the heart's action may be fairly inferred. Should the change be very decided and permanent, the cause must nearly always be sought in the valves themselves; but if slight and temporary, it is often a mere muscular or functional disturbance, not dependant on organic disease.

The sounds of the heart may be altered in several ways: they may be changed in character, or merely diminished or increased in intensity. The alteration of the sounds may be limited to a slight harshness, or the natural tone may be totally changed; these characters, however, differ only in degree, and not in any really important respects.

The first sound is most frequently altered. When simply increased in loudness, it depends either upon a temporary condition of the heart, that is, a simple febrile movement or nervous action, in which case the sound will after a time subside to the natural state, or it arises from a hardening of the muscular structure of the heart, perhaps conjoined with slight obstruction of the semilunar valves. In the latter case the increased loudness, or, to use an equivalent expression, the roughness of the sound may continue for a very long period.

If the roughness is increased, it passes into the bellows or rasping sound. The former of these is less marked than the latter, but a bellows sound may be defined, and it is generally described, as a prolonged and purring sound, generally heard in the first sound of the heart, and therefore produced chiefly by muscular contraction, although it may arise from alterations at the auriculo-ventricular valves, and then it occurs during the diastole of the heart. Like all sounds, it is much more easy to point out than to describe in words. As a short definition, the term bellows sound, which is given to it, from resembling the sound produced by blowing strongly in a bellows, is probably as good a description as any other. The bellows sound is often produced by simple nervous disorder of the heart, especially in those cases in which it is connected with anemia or chlorosis, and it then is very loud and almost musical in its tone; and far from being confined to the heart, it may be distinguished along the whole of the large arteries, especially the carotids and subclavian, by applying the stethoscope opposite to them. The pressure of the stethoscope has probably some influence in favouring the production of the sound at the carotids, but is insufficient to account for it; so that we are obliged to ascribe it to the peculiar motion impressed upon the thin and watery blood by the spasmodic action of the heart. When the bellows sound depends either on a hypertrophied ventricle urging the blood very rapidly through a narrow or non-dilated semilunar valve, or driving it back through a dilated auriculo-ventricular opening, it is more persistant, more uniform, and is less musical, but more harsh than when it arises from a mere nervous disorder; the same character is found when the sound is heard during the diastole from regurgitation through the semilunar, or contraction of the auriculo-ventricular valves. Still it is in many cases difficult to distingush between the bellows sound of mere functional disorder and that dependant upon organic disease, unless there are some other signs of a permanent lesion.

The rasping sound of the heart is much rougher than the bellows sound, and is tolerably well described by the term which designates it—resembling the sound of a rasp forced through soft wood more than any other sound. It never depends upon simple functional disorder of the heart, but arises from some actual obstruction to the circulation of the blood, seated at the orifices of the heart, and therefore dependant upon changes in the valves. It may arise from acute as well as chronic disease; when the obstruction is acute, it is the result of endocarditis, and the thickening of the valves is partly caused by depositions of lymph, and partly by thickening of the fibrous tissue, which forms the body of the valve, and, as the inflammation declines, the sound will gradually increase, provided the morbid product has not become completely organised, in which case the rasping sound may be permanent. The sign is scarcely ever heard during the diastole of the heart, for it requires a considerable force in the current of blood, and the act of dilatation is rarely sufficient to produce this at the valvular orifices. Such is not, however, the case when the aorta is much dilated, for the reflux of the blood is almost as powerful as its forward current, and the rasping is therefore double, like the forward and backward motion of a saw rather than of a rasp; hence it is then called the sawing (bruit de scie) instead of the rasping sound, and is one of the best diagnostic characters of aneurism of the aorta. The double movement and the accompanying sound are so peculiar that it can scarcely be mistaken for any other sign. The saw-sound is heard also at times when the mitral valve or the tricuspid is much altered, so as to destroy its functions and convert the auriculo-ventricular opening into a rough passage for the blood.

